# Integrated Retrospective and Post‐GWAS Analyses Reveal Mechanisms Linking Nightly Short Sleep to Asthma

**DOI:** 10.1002/brb3.71001

**Published:** 2025-10-15

**Authors:** Chong Fu, Wei Xu, Yanping Zhang

**Affiliations:** ^1^ Department of Gastroenterology Anqing Municipal Hospital Anqing Anhui PR China

**Keywords:** asthma, causal inference, neuroimmunology, nightly short sleep, pleiotropy

## Abstract

**Objectives:**

This study employs a dual‐pronged approach, integrating retrospective cohort analysis with genetic methodologies, to elucidate the causal role of nightly short sleep in the pathogenesis of asthma and to unravel its underpinning biological mechanisms.

**Methods:**

This study employed a dual‐evidence framework for a comprehensive analysis. The retrospective cohort component utilized data from 6770 participants in the China Health and Retirement Longitudinal Study (CHARLS) to assess the dose‐response relationship and risk thresholds for asthma incidence via multivariable logistic regression, restricted cubic splines, and segmented regression models. The genetic analysis component integrated large‐scale Genome‐Wide Association Studies (GWAS) data for nightly short sleep from the UK Biobank and for asthma from the FinnGen biobank. A suite of methodologies, including Linkage Disequilibrium Score Regression (LDSC), High‐Definition Likelihood (HDL), Pleiotropic Analysis under Composite Null Hypothesis, (PLACO), Colocalization (COLOC), and Summary‐data‐based Mendelian Randomization (SMR), was employed to evaluate genetic correlations and identify shared loci. To infer causality, this study applied a battery of advanced, robust MR models—including Mendelian Randomization‐Clustering (MR‐Clust), Maximum Likelihood Mendelian Randomization (MRcML), Contamination Mixture (ConMix), and CAUSE—to systematically correct for and evaluate the influence of horizontal pleiotropy.

**Results:**

The cohort regression analysis revealed that increased sleep duration confers a significant protective effect against asthma (Model 3: OR = 0.83, 95% CI 0.75–0.92). A pronounced dose‐response trend was observed (P for trend < 0.0004), wherein longer sleep corresponded to lower risk. Further analysis with restricted cubic splines confirmed a U‐shaped, nonlinear relationship, identifying a risk inflection point at 7.5 h of sleep. Subgroup analyses indicated that this protective effect was robust across diverse age, gender, and lifestyle strata, with no significant interaction effects detected (all P‐interaction > 0.05). At the genetic level, a significant positive genetic correlation was established between nightly short sleep and asthma (LDSC rg = 0.257; HDL rg = 0.247, with *p* < 0.001 for both). Colocalization analysis identified three shared causal loci (rs6939576, rs13107325, and rs205024) with distinct protein‐altering and regulatory functions. Subsequent SMR analysis identified three shared causal genes—TBX6, ABT1, and YPEL3—with directionally consistent effects. Consistent evidence from multiple analytical models—including MR‐cML (*β* = 0.927, *p* = 0.0009), ConMix (*β* = 1.585, *p* = 0.0006), and CAUSE (favoring the causal model, ΔELPD = −3.3; causal effect *γ* = 0.54)—supports the conclusion that genetically predicted nightly short sleep is a causal factor for an increased risk of asthma.

**Conclusion:**

This research provides compelling evidence substantiating nightly short sleep as a causal risk factor for asthma, with genetically predicted nightly short sleep significantly elevating disease risk.

## Graphical abstract

This study investigates the potential link between short sleep and asthma through two approaches.

**Retrospective Cohort Study**: Utilizing data from the China Health & Retirement Study, this study explored the association between short nighttime sleep and incident asthma by adjusting for multiple baseline factors.

**Post‐Genome‐Wide Association Study**: Using GWAS summary data from large biobanks, this study first explored the genetic link through genetic correlation and identification of pleiotropic loci. Subsequently, a Mendelian Randomization analysis was performed to evaluate the causal effect. The reliability of the results was verified through multiple sensitivity analyses and advanced correction methods.

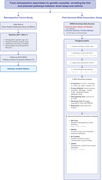

## 1 Introduction

Asthma, a pervasive chronic respiratory ailment, imposes a substantial global health burden across all age demographics, characterized by high morbidity alongside low rates of mortality and remission. According to the 2019 Global Burden of Disease study, asthma was responsible for 461,100 deaths and 21.55 million disability‐adjusted life years, with years of life lost due to premature mortality constituting over half of this total burden (Shin et al. 2023). Notably in China, while the mortality rate is not among the world's highest, its immense population base resulted in the nation accounting for 5.37% of all global asthma deaths in 2019, ranking it third worldwide (Huang et al. 2019).

Epidemiological investigations have extensively documented an association between sleep duration and asthma risk. Studies based on NHANES data, for instance, have revealed a U‐shaped correlation between sleep duration and symptoms such as coughing and dyspnea, with both short (≤ 5 h) and long (≥ 9 h) sleep durations being linked to diminished pulmonary function (Yang et al. 2019). However, such observational research, often analyzing sleep as a categorical variable and thus overlooking the effects of its continuous nature, is incapable of substantiating causality or precisely defining a safe range of sleep. More critically, a conspicuous lacuna persists in the scientific understanding of potential bidirectional causality and shared genetic mechanisms between nightly short sleep and asthma. Although prior research has attempted to explore this nexus using Mendelian randomization (Ni et al. 2025), the conclusions have been rendered unreliable, hampered by insufficient statistical power and the confounding presence of pleiotropic instruments that violate the method's core assumptions, thereby underscoring the urgent need for more rigorous inquiry in this domain.

**FIGURE 1:**
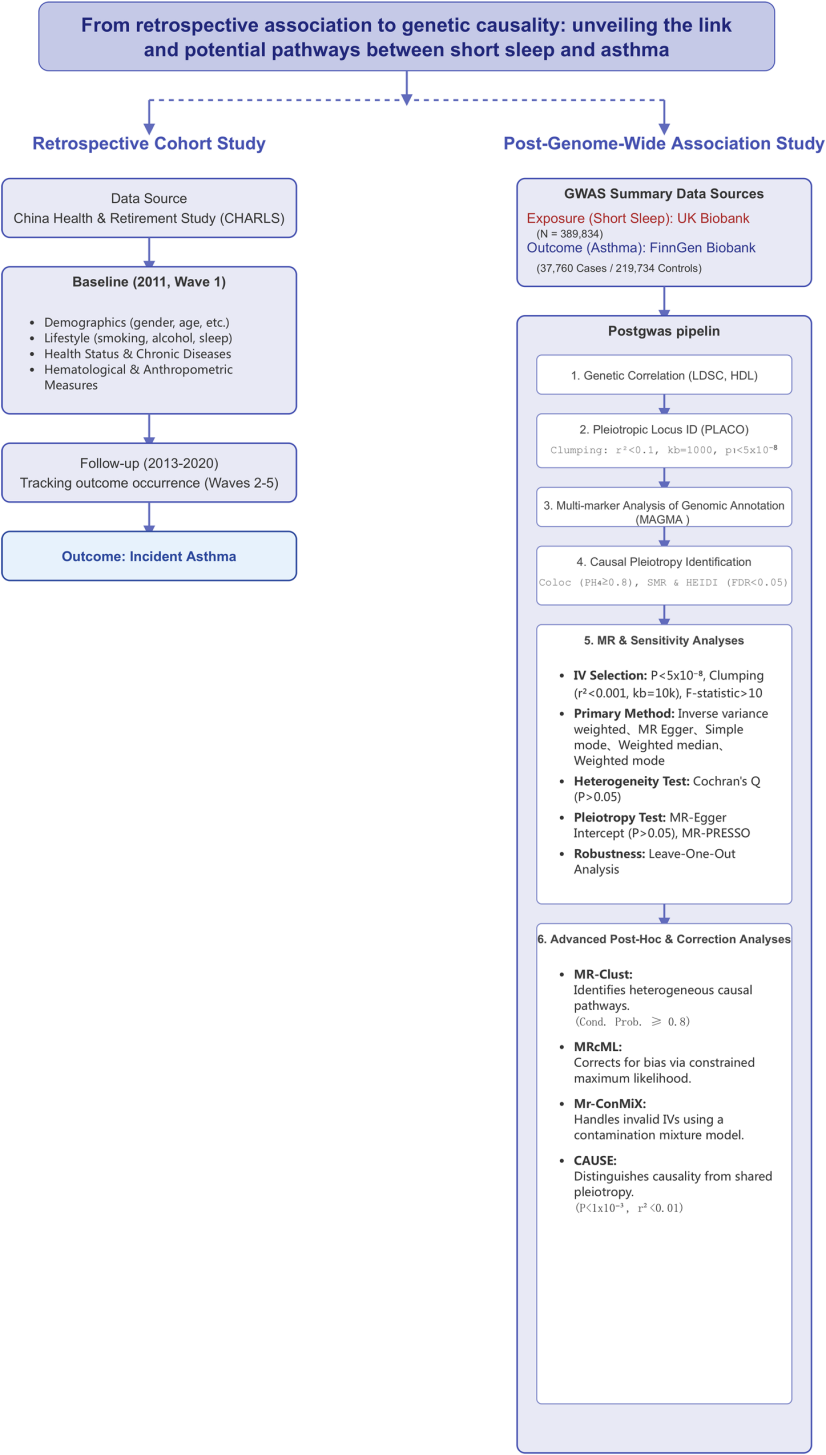
Flow diagram.

This study, therefore, endeavors to conduct a more systematic and profound exploration of this relationship. We will first employ restricted cubic spline (RCS) models to meticulously delineate the continuous dose‐response relationship between sleep duration and asthma incidence, with the objective of identifying the sleep threshold associated with the lowest risk. Building upon this, we will integrate the latest data from Genome‐Wide Association Studies (GWAS) to comprehensively dissect the shared genetic architecture between nightly short sleep and asthma. This will be achieved by applying a diverse suite of analytical techniques, including Linkage Disequilibrium Score Regression (LDSC), High‐Definition Likelihood (HDL), Pleiotropic Analysis under Composite Null Hypothesis (PLACO), Colocalization (COLOC), Multi‐Marker Analysis of Genomic Annotation (MAGMA), and Summary‐data‐based Mendelian Randomization (SMR). Finally, any putative causal association will be rigorously validated using multiple robust Mendelian randomization methods, such as Mendelian Randomization‐Clustering (MR‐Clust), constrained Maximum Likelihood Mendelian Randomization (MR‐cML), and the Contamination Mixture (ConMix) model. The overarching aim of this research is to furnish a more substantial scientific foundation for the precision prevention and control of asthma in middle‐aged and older populations, with the overall study design summarized in Figure 1.

## 2 Methods

### 2.1 Baseline Data Collection

This investigation was designed as a retrospective cohort study, utilizing data from the China Health and Retirement Longitudinal Study (CHARLS), a nationwide longitudinal database. Leveraging prospectively collected data from CHARLS, the study cohort was established with participants who were free of asthma at the baseline survey in 2011 (Wave 1). These individuals were subsequently monitored for outcome incidence through follow‐up surveys conducted in 2013 (Wave 2), 2015 (Wave 3), 2018 (Wave 4), and 2020 (Wave 5).

Baseline data encompassed a comprehensive array of variables, including: (1) Demographic characteristics: gender, age, marital status, educational attainment, and residence type (urban/rural); (2) Lifestyle factors: smoking history, alcohol consumption patterns, and nightly sleep duration; (3) Health status: prevalent asthma; (4) Biochemical and anthropometric measures: a panel of hematological markers (white blood cell count, C‐reactive protein, platelet count, blood urea nitrogen, creatinine, glucose, uric acid, total cholesterol, triglycerides, high‐density lipoprotein cholesterol, and low‐density lipoprotein cholesterol) and Body mass index (BMI); and (5) Comorbidities: presence of chronic conditions such as hypertension, diabetes, and cancer. The diagnosis of chronic diseases was ascertained through self‐report, based on participants' affirmative responses to the question, “Has a doctor ever told you that you have [chronic disease]?”

### 2.2 Follow‐up and Outcome Ascertainment

The primary outcome variable for this study was incident asthma. The diagnosis of asthma during the follow‐up period was determined through a health status questionnaire, wherein participants were identified as having incident asthma if they responded affirmatively to the question, “Has a doctor ever told you that you have asthma?”

### 2.3 Data Sources and Instrumental Variable Selection

GWAS summary statistics for nightly short sleep duration were procured from the UK Biobank, comprising a sample size of 411,934 individuals. Correspondingly, genetic summary data for asthma were obtained from the FinnGen biobank consortium's GWAS, encompassing 37,760 cases and 219,734 controls of European ancestry.

### 2.4 Genetic Correlation

LDSC was employed to quantify heritability by interrogating the relationship between the effect sizes of individual Single Nucleotide Polymorphism (SNPs) and their linkage disequilibrium (LD) scores from GWAS summary data, thereby facilitating the assessment of shared genetic architecture between traits (Bulik‐Sullivan et al. 2015). In contrast, the HDL method, which is predicated on a maximum likelihood estimation framework under a multivariate normal distribution, obviates the need for specific assumptions regarding the distribution of genetic effects. This approach theoretically affords more precise discrimination between causal and non‐causal variants, yielding more robust estimations of genetic correlation (Ning et al. 2020). In the present study, both methodologies were utilized in a cross‐validation framework to ensure the reliability of the findings.

### 2.5 Identification of Pleiotropic Loci

The PLACO method was employed to identify pleiotropic loci influencing both traits by testing a composite null hypothesis—that a given genetic locus is associated with zero or one trait. We extended its application to discover pleiotropic associations at the gene level. PLACO evaluates the association of a gene with two traits via *Z*‐statistics and delineates the composite null hypothesis into three sub‐hypotheses: association with the first trait but not the second, association with the second trait but not the first, or association with neither trait. The alternative hypothesis, corresponding to a pleiotropic association, posits that the gene is associated with both traits (Ray and Chatterjee 2020). A significance threshold was established at *p* < 5 × 10^−8^, below which a gene was classified as a pleiotropic SNP for both traits. Subsequently, PLINK clumping analysis was applied to derive independent SNVs, using the parameters –clump‐p1 5 × 10^−8^, –clump‐p2 1 × 10^−5^, –clump‐r2 0.1, and –clump‐kb 1000 (Purcell et al. 2007).

### 2.6 Identification of Pleiotropic Genes

The MAGMA methodology ascertains the association between human protein‐coding genes and a specific phenotype by aggregating the association *p*‐values of intragenic variants from GWAS summary data. In this investigation, we established the following criteria for a positive finding: To mitigate the burden of multiple hypothesis testing, the resultant MAGMA *p*‐values for each phenotype were subjected to a False Discovery Rate (FDR) correction, scaled to the total number of genes analyzed for the trait. An FDR‐adjusted *q*‐value of < 0.05 served as the threshold for delineating significantly associated genes. Consequently, a gene was defined as pleiotropic if it exhibited a significant association with both traits and was physically situated within or spanned a predefined pleiotropic locus (Groenewoud et al. 2022).

### 2.7 Enrichment Analysis of Biological Pathways

Utilizing the clusterProfiler package, we conducted Gene Ontology (GO) and Kyoto Encyclopedia of Genes and Genomes (KEGG) enrichment analyses on pleiotropic genes. The GO analysis encompassed three domains: Biological Process (BP), Molecular Function (MF), and Cellular Component (CC). For visualization, the top 10 entries from Molecular Function (MF), Biological Process (BP), and Cellular Component (CC) were selected, while the top 20 KEGG pathways were chosen for visual representation.

### 2.8 Identification of Causal Pleiotropic Loci

To ascertain whether the association signals for short‐duration sleep and asthma shared a common genetic etiology, colocalization analysis was conducted utilizing the coloc R package. For SNPs associated with both phenotypes, all variants within a 500‐kilobase window flanking the index SNV were interrogated. Subsequently, the posterior probability of Hypothesis 4 (H4), which posits a single, shared causal variant for both traits, was calculated. A posterior probability (PPH4) of 0.8 or greater was considered strong evidence of colocalization, whereas a PPH4 between 0.5 and 0.8 was deemed indicative of moderate evidence (Giambartolomei et al. 2014). Putative causal variants were then subjected to functional annotation using the CADD and RegulomeDB frameworks. Each SNP was assigned a CADD score (Kircher et al. 2014) to quantify its predicted deleteriousness, with scores exceeding 10 signifying a threshold for potential pathogenicity. The RegulomeDB score (Kircher et al. 2014), a categorical metric derived from eQTL and chromatin mark data, ranks variants from 1a to 7, wherein lower scores denote a greater probability of regulatory function.

### 2.9 Identification of Causal Pleiotropic Genes

To elucidate whether the genetic effects of SNPs on a target phenotype are mediated through gene expression, we employed an SMR approach, leveraging summary statistics from GWAS. All analyses were executed using the SMR software package, a process entailing the rigorous harmonization of alleles between the GWAS and expression quantitative trait loci (eQTL) datasets. To differentiate true pleiotropy from confounding effects arising from linkage disequilibrium, we further implemented a Heterogeneity in Dependent Instruments (HEIDI) test. A HEIDI test *p*‐value below 0.05 was interpreted as evidence that the observed association between gene expression and the phenotype likely originates from linkage effects rather than genuine pleiotropy, thus leading to the exclusion of the implicated gene from subsequent consideration (Zhu et al. 2016).

### 2.10 Prediction of Candidate Therapeutics

To identify potential pharmacological agents for the treatment of short‐duration sleep and asthma, the present study utilized the Drug Signatures Database (DSigDB; https://dsigdb.tanlab.org/DSigDBv1.0/) as the source for curated sets of drug‐target genes. We procured the functionally classified drug‐target gene sets (“Categories” data, as updated in February 2022) from this repository (Song 2023). Subsequently, these gene sets were subjected to an enrichment analysis with the objective of delineating drug categories that were significantly associated with our genes of interest. For this analysis, an adjusted *p*‐value of less than 0.05 was established as the criterion for statistically significant enrichment.

### 2.11 Mendelian Randomization Analysis

To curate a set of instrumental variables (IVs) for nightly short sleep duration, SNPs were selected based on a genome‐wide significance threshold (*p* < 5 × 10^−8^). To mitigate the influence of linkage disequilibrium, a clumping procedure was applied with parameters set to kb = 10,000 and *r* ^2^ < 0.001. The strength of the selected instruments was assessed by calculating the F‐statistic, where a value exceeding 10 was considered indicative of a low risk of weak instrument bias, thereby satisfying the relevance assumption. The *F*‐statistic was calculated as *F* = [(*N*−K−1)/K] × [*R* ^2^/(1−*R* ^2^)], with *N* representing the exposure sample size, K the number of IVs, and *R* ^2^ the proportion of variance in the exposure explained by the IVs (Burgess et al. 2011). The analysis ensured no sample overlap between the exposure and outcome GWAS, and all participants were of European ancestry. The causal effect of nightly short sleep duration on asthma was primarily estimated using the random‐effects inverse‐variance weighted (IVW) method. This analysis relies on three core assumptions: (1) the relevance assumption, where IVs are robustly associated with the exposure; (2) the independence assumption, where IVs are not associated with any confounders of the exposure‐outcome relationship; and (3) the exclusion‐restriction assumption, where IVs affect the outcome exclusively through the exposure (Davies et al. 2018). To complement the primary analysis and enhance the robustness of our findings, we employed four additional MR methods: MR‐Egger, weighted median, simple mode, and weighted mode. A *p*‐value of less than 0.05 was considered statistically significant for the causal estimates, and all statistical tests were two‐sided. The results are presented as odds ratios (ORs).

### 2.12 Sensitivity Analyses

Several sensitivity analyses were conducted to evaluate the validity of the MR assumptions. Heterogeneity among the IVs was assessed using Cochran's *Q* statistic for both the IVW and MR‐Egger methods, with a *p*‐value > 0.05 indicating no significant heterogeneity. Directional horizontal pleiotropy was evaluated using the intercept from the MR‐Egger regression and further examined using the MR‐PRESSO (Pleiotropy RESidual Sum and Outlier) test; a *p*‐value > 0.05 for these tests suggests the absence of significant pleiotropy. Furthermore, a “leave‐one‐out” analysis was performed to ensure that the overall causal estimate was not disproportionately influenced by any single SNP. All statistical analyses were conducted using R software (version 4.2.2) with the “TwoSampleMR” (Hemani et al., 2018) and “MRPRESSO” (Verbanck et al., 2018) packages.

### 2.13 MR‐Clust Analysis of nightly short sleep on Asthma

To investigate potential heterogeneity in causal pathways, a cluster analysis was conducted using the MR‐Clust method. This approach, which is predicated on MR causal effect estimates, automatically determines the optimal number of clusters via the Bayesian Information Criterion. It partitions genetic variants into “substantive clusters” characterized by similar causal effects, while simultaneously delineating “null clusters” (comprising non‐causal variants) and “junk clusters” (containing unclassifiable variants). To ensure the fidelity of the clusters, a conditional probability threshold of ≥ 0.8 and a minimum of four variants per cluster were required (Foley et al. 2021).

### 2.14 MRcML

The MRcML method was employed to correct for pleiotropic bias in the instrumental variables. This approach simultaneously estimates the causal effect (*θ*) and variant‐specific pleiotropic effects (α_i) by maximizing a joint likelihood function under a sparsity constraint (Σ|α_i| ≤ *τ*). The strength of this constraint, *τ*, is adaptively selected using the Bayesian Information Criterion (BIC), which significantly enhances the robustness of the estimation in scenarios involving complex pleiotropy (Xue et al. 2021).

### 2.15 Mr_ConMiX

The core principle of the Mr_ConMiX method is the application of a “contamination mixture model” to address the pervasive issue of horizontal pleiotropy in MR analyses. This phenomenon occurs when certain IVs influence the outcome through pathways independent of the exposure, leading to biased causal inference. Mr_ConMiX postulates that the set of IVs constitutes a mixture of “valid” and “invalid” (i.e., pleiotropic) instruments. By maximizing a likelihood function that accommodates the distributions of both instrument types, the model concurrently estimates the true causal effect while identifying potentially invalid instruments. This method is particularly powerful when the ‘plurality valid’ assumption holds, thereby furnishing more reliable and accurate causal estimates than can be achieved with conventional MR methods, especially in the presence of some invalid instruments (Burgess et al. 2020).

### 2.16 CAUSE

CAUSE was utilized to judiciously disentangle veritable causal effects from spurious associations arising from shared genetic factors. This is achieved through a unique Bayesian model comparison framework. The model integrates a large number of pruned SNPs (selected with a *p*‐value threshold of 1 × 10^−3^, *r* ^2^ < 0.01, and a clumping distance of 500 kb) and implements a Bayesian model comparison to differentiate between a ‘causal’ model and a ‘sharing’ model (one characterized by correlated pleiotropy) (Morrison et al. 2020).

### 2.17 Statistical Analysis

Descriptive statistics were generated for the study population. Continuous variables were expressed as means and standard deviations, while categorical variables were presented as frequencies and percentages. Inter‐quartile differences in baseline variables were compared using one‐way analysis of variance (ANOVA), the Kruskal–Wallis *H* test, or the chi‐square test, as appropriate. To meticulously examine the association between sleep duration and asthma risk, a series of sequentially adjusted multivariable logistic regression models was constructed. Model 1 represented the crude, unadjusted association. Model 2 was adjusted for demographic variables, including gender, age, and marital status. Model 3 was further adjusted for social and behavioral factors (educational attainment, urban/rural residence), environmental exposures (use of polluting fuels for heating/cooking), and inflammatory markers (C‐reactive protein). To explore potential non‐linearity in the relationship, a Generalized Additive Model (GAM) incorporating smoothing spline functions was employed for curve fitting. The presence of potential threshold effects was subsequently interrogated using a segmented regression model. Furthermore, stratified subgroup analyses were conducted across various dimensions (including gender, age strata, and environmental exposures), coupled with tests for interaction, to systematically evaluate potential effect modification by different population characteristics. All data utilized in this study were derived from previously published research or publicly accessible GWAS databases, for which ethical approval and informed consent had already been obtained. Consequently, no separate ethical approval was required for the present analysis.

## 3 Results

### 3.1 Baseline Characteristics

Adhering to the specified inclusion and exclusion criteria, a final cohort comprising 6770 participants was enrolled in this study. The mean age of the participants was 59 ± 9.10 years, with the cohort consisting of 3660 males (54.06%) and 3110 females (45.94%). Upon stratifying the cohort by quartiles of sleep duration, an analysis of the demographic and clinical characteristics revealed statistically significant inter‐group differences (Q1–Q4) across several variables. These included key demographic features (age, gender, urban/rural residence), metabolic indicators (body mass index, creatinine, high‐density lipoprotein), and environmental‐behavioral factors (smoking, alcohol consumption, use of clean fuel). Notably, individuals in the highest quartile of sleep duration (Q4), representing the longest sleepers, exhibited a greater proportion of females, a lower prevalence of rural residency, and a higher rate of clean cooking fuel utilization when compared to the other groups (Table 1). This constellation of baseline characteristics suggests that factors such as social support and environmental resources may be conducive to prolonged sleep duration.

**TABLE 1:** Baseline characteristics of the study population.

Characteristic	Overall	Q0	Q1	Q2	Q3	*p*‐value	SMD
*N*	6770	1068	2276	1359	2067		
Age	58.00 (52.00–65.00)	60.00 (54.00–68.00)	58.00 (53.00–65.00)	57.00 (50.00–63.00)	58.00 (51.00–65.00)	< 0.001	0.217
Body mass index	23.25 (20.98–25.96)	22.50 (20.38–25.29)	23.40 (21.05–26.07)	23.64 (21.38–26.13)	23.16 (20.99–26.01)	< 0.001	0.084
White blood cell count	5.90 (4.90–7.20)	6.00 (4.90–7.40)	5.93 (4.90–7.10)	5.90 (4.90–7.10)	5.90 (5.00–7.20)	0.825	0.050
Platelet count	208.00 (163.00–255.00)	207.00 (163.00–255.00)	208.00 (164.00–255.00)	209.00 (167.00–254.00)	207.00 (161.00–258.00)	0.885	0.009
Blood urea nitrogen	15.06 (12.52–18.12)	15.36 (12.74–18.47)	14.97 (12.49–18.04)	14.99 (12.49–18.01)	15.04 (12.46–17.93)	0.212	0.049
Glucose	102.60 (94.68–113.58)	102.24 (94.68–112.68)	102.96 (95.22–113.94)	102.60 (94.14–113.58)	102.60 (94.86–113.58)	0.451	0.015
Creatinine	0.76 (0.66–0.88)	0.75 (0.64–0.86)	0.75 (0.66–0.88)	0.76 (0.67–0.88)	0.77 (0.66–0.89)	0.043	0.007
Total cholesterol	189.82 (167.01–214.95)	190.59 (167.69–216.59)	189.82 (167.01–215.72)	190.59 (168.75–215.72)	188.66 (165.85–212.63)	0.092	0.049
Triglycerides	107.08 (76.11–156.65)	107.08 (75.22–152.22)	107.08 (77.00–159.30)	107.08 (74.34–158.41)	107.08 (76.11–155.76)	0.882	0.025
HDL triglycerides	48.71 (40.21–59.44)	50.64 (40.98–60.70)	48.90 (39.82–59.15)	48.33 (39.82–58.76)	48.33 (39.82–58.76)	0.002	0.068
LDL triglycerides	114.05 (93.56–136.86)	114.43 (92.30–135.41)	113.66 (93.17–138.02)	116.37 (94.72–138.60)	113.66 (94.72–135.12)	0.279	0.022
C‐reactive protein	1.03 (0.55–2.11)	1.05 (0.54–2.22)	1.03 (0.57–2.12)	1.03 (0.54–2.21)	1.03 (0.54–1.97)	0.847	0.017
Uric acid	4.27 (3.55–5.12)	4.29 (3.51–5.13)	4.29 (3.56–5.12)	4.22 (3.57–5.08)	4.27 (3.56–5.14)	0.786	0.017
Nightly sleep duration	7.00 (5.00–8.00)	4.00 (3.00–4.00)	6.00 (5.00–6.00)	7.00 (7.00–7.00)	8.00 (8.00–9.00)	< 0.001	4.273
Gender						< 0.001	0.117
Female	3660 (54.06%)	664 (62.17%)	1227 (53.91%)	711 (52.32%)	1058 (51.19%)		
Male	3110 (45.94%)	404 (37.83%)	1049 (46.09%)	648 (47.68%)	1009 (48.81%)		
Smoking status						0.027	0.062
No	4715 (69.65%)	781 (73.13%)	1593 (69.99%)	924 (67.99%)	1417 (68.55%)		
Yes	2055 (30.35%)	287 (26.87%)	683 (30.01%)	435 (32.01%)	650 (31.45%)		
Alcohol Consumption						0.005	0.075
No	4532 (66.94%)	759 (71.07%)	1524 (66.96%)	873 (64.24%)	1376 (66.57%)		
Yes	2238 (33.06%)	309 (28.93%)	752 (33.04%)	486 (35.76%)	691 (33.43%)		
Hypertension						0.708	0.022
No	4950 (73.12%)	769 (72.00%)	1661 (72.98%)	992 (72.99%)	1528 (73.92%)		
Yes	1820 (26.88%)	299 (28.00%)	615 (27.02%)	367 (27.01%)	539 (26.08%)		
Diabetes						0.087	0.045
No	6357 (93.90%)	1008 (94.38%)	2138 (93.94%)	1257 (92.49%)	1954 (94.53%)		
Yes	413 (6.10%)	60 (5.62%)	138 (6.06%)	102 (7.51%)	113 (5.47%)		
Residence						< 0.001	0.111
Urban	2396 (35.39%)	317 (29.68%)	870 (38.22%)	523 (38.48%)	686 (33.19%)		
Rural	4374 (64.61%)	751 (70.32%)	1406 (61.78%)	836 (61.52%)	1381 (66.81%)		
Clean fuel for heating						< 0.001	0.118
No	2114 (31.23%)	257 (24.06%)	753 (33.08%)	463 (34.07%)	641 (31.01%)		
Yes	4656 (68.77%)	811 (75.94%)	1523 (66.92%)	896 (65.93%)	1426 (68.99%)		
Clean Fuel for Cooking						< 0.001	0.148
No	2841 (41.96%)	355 (33.24%)	1016 (44.64%)	630 (46.36%)	840 (40.64%)		
Yes	3929 (58.04%)	713 (66.76%)	1260 (55.36%)	729 (53.64%)	1227 (59.36%)		

### 3.2 Univariate and Multivariate Analyses

The univariate analysis identified several factors associated with the risk of asthma. A significantly higher risk was observed with increasing age (OR = 1.03, 95% CI: 1.02–1.04, *p* < 0.01) and among rural residents compared to their urban counterparts (OR = 1.39, 95% CI: 1.11–1.75, *p* < 0.01). The non‐use of clean energy for heating (OR = 1.34, 95% CI: 1.05–1.72, *p* = 0.02) and cooking (OR = 1.42, 95% CI: 1.13–1.77, *p* < 0.01) were also linked to an elevated risk. Conversely, protective factors included a higher level of educational attainment (OR = 0.54, 95% CI: 0.35–0.82, *p* < 0.01) and each additional hour of nightly sleep duration (OR = 0.89, 95% CI: 0.84–0.94, *p* < 0.01). Among inflammatory markers, both white blood cell count (OR = 1.05, 95% CI: 1.00–1.11, *p* = 0.05) and C‐reactive protein (OR = 1.01, 95% CI: 1.00–1.02, *p* = 0.04) exhibited a marginal positive association. Factors such as gender, alcohol consumption, and LDL cholesterol levels did not reach statistical significance (*p* ≥ 0.05) (Table 2). In multivariate analysis, nightly sleep duration remained a significant protective factor; each additional hour of sleep reduced the risk of asthma (OR = 0.91, 95% CI: 0.86–0.96, *p* < 0.01), while other factors did not show a significant association with asthma risk (Table 3).

**TABLE 2:** The results of univariate analysis.

Characteristic	Statistics	Effect value (95% CI)	*p*‐value
Age	59.0 ± 9.1	1.03 (1.02, 1.04)	< 0.01
Gender: Female	3660 (54.1%)	Ref	
Gender: Male	3110 (45.9%)	1.07 (0.87, 1.32)	0.50
Smoking status: No	4715 (69.6%)	Ref	
Smoking status: Yes	2055 (30.4%)	1.19 (0.95, 1.48)	0.13
Alcohol consumption: No	4532 (66.9%)	Ref	
Alcohol consumption: Yes	2238 (33.1%)	0.88 (0.70, 1.10)	0.26
Hypertension: No	4950 (73.1%)	Ref	
Hypertension: Yes	1820 (26.9%)	1.06 (0.84, 1.34)	0.60
Diabetes: No	6357 (93.9%)	Ref	
Diabetes: Yes	413 (6.1%)	0.81 (0.50, 1.29)	0.37
Residence: No	2396 (35.4%)	Ref	
Residence: Yes	4374 (64.6%)	1.39 (1.11, 1.75)	< 0.01
Clean fuel for heating: No	2114 (31.2%)	Ref	
Clean fuel for heating: Yes	4656 (68.8%)	1.34 (1.05, 1.69)	0.02
Clean fuel for cooking: No	2841 (42.0%)	Ref	
Clean fuel for cooking: Yes	3929 (58.0%)	1.42 (1.14, 1.77)	<0.01
Body mass index	23.9 ± 11.4	1.00 (1.00, 1.01)	0.09
White blood cell count	6.2 ± 1.8	1.05 (1.00, 1.11)	0.05
Platelet count	213.1 ± 75.9	1.00 (1.00, 1.00)	0.19
Blood urea nitrogen	15.6 ± 4.5	1.01 (0.99, 1.04)	0.21
Glucose	110.6 ± 35.3	1.00 (1.00, 1.00)	0.88
Creatinine	0.8 ± 0.2	1.11 (0.78, 1.60)	0.56
Total cholesterol	192.9 ± 37.9	1.00 (1.00, 1.00)	0.13
Triglycerides	133.2 ± 95.1	1.00 (1.00, 1.00)	0.41
HDL triglycerides	50.7 ± 15.2	1.00 (0.99, 1.01)	0.72
LDL triglycerides	116.4 ± 34.7	1.00 (0.99, 1.00)	0.08
C‐reactive protein	2.6 ± 6.8	1.01 (1.00, 1.02)	0.04
Uric acid	4.4 ± 1.2	1.04 (0.96, 1.13)	0.29
Nightly sleep duration	6.4 ± 1.9	0.89 (0.84, 0.94)	< 0.01

**TABLE 3:** The results of Multivariate analysis.

Characteristic	Statistics	Effect value (95% CI)	*p*‐value
Age	59.0 ± 9.1	1.02 (1.00, 1.04)	0.11
Gender: Female	3660 (54.1%)	Ref	
Gender: Male	3110 (45.9%)	1.12 (0.89, 1.40)	0.33
Smoking status: No	4715 (69.6%)	Ref	
Smoking status: Yes	2055 (30.4%)	1.19 (0.95, 1.49)	0.12
Alcohol consumption: No	4532 (66.9%)	Ref	
Alcohol consumption: Yes	2238 (33.1%)	0.92 (0.73, 1.16)	0.47
Hypertension: No	4950 (73.1%)	Ref	
Hypertension: Yes	1820 (26.9%)	0.99 (0.78, 1.25)	0.93
Diabetes: No	6357 (93.9%)	Ref	
Diabetes: Yes	413 (6.1%)	0.81 (0.50, 1.31)	0.39
Residence: No	2396 (35.4%)	Ref	
Residence: Yes	4374 (64.6%)	1.21 (0.94, 1.55)	0.14
Clean fuel for heating: No	2114 (31.2%)	Ref	
Clean fuel for heating: Yes	4656 (68.8%)	1.03 (0.78, 1.36)	0.84
Clean fuel for cooking: No	2841 (42.0%)	Ref	
Clean fuel for cooking: Yes	3929 (58.0%)	1.21 (0.94, 1.57)	0.14
Body mass index	23.9 ± 11.4	1.00 (1.00, 1.01)	0.05
White blood cell count	6.2 ± 1.8	1.04 (0.99, 1.10)	0.12
Platelet count	213.1 ± 75.9	1.00 (1.00, 1.00)	0.25
Blood urea nitrogen	15.6 ± 4.5	1.01 (0.98, 1.03)	0.61
Glucose	110.6 ± 35.3	1.00 (1.00, 1.00)	1.00
Creatinine	0.8 ± 0.2	1.09 (0.74, 1.60)	0.68
Total cholesterol	192.9 ± 37.9	1.00 (0.99, 1.00)	0.10
Triglycerides	133.2 ± 95.1	1.00 (1.00, 1.00)	0.86
HDL triglycerides	50.7 ± 15.2	1.00 (0.99, 1.01)	0.72
LDL triglycerides	116.4 ± 34.7	1.00 (0.99, 1.00)	0.05
C‐reactive protein	2.6 ± 6.8	1.01 (1.00, 1.02)	0.08
Uric acid	4.4 ± 1.2	1.05 (0.97, 1.14)	0.26
Nightly sleep duration	6.4 ± 1.9	0.91 (0.86, 0.96)	<0.01

### 3.3 Regression Analysis of the Association Between Sleep Duration and Asthma Incidence

In the unadjusted analysis (Model 1), a significant inverse association was observed between sleep duration and the risk of incident asthma (OR = 0.80, 95% CI: 0.72–0.88, *p* < 0.0001). This association retained its statistical significance following sequential adjustment for potential confounding variables in Model 2 (OR = 0.83, 95% CI: 0.75–0.92, *p* = 0.0002) and in the fully adjusted Model 3 (OR = 0.83, 95% CI: 0.75–0.92, *p* = 0.0003). Furthermore, a consistent and significant dose‐response relationship was evident across all three models (P for trend < 0.0001), demonstrating that the risk of asthma progressively decreased with each increasing quartile of sleep duration (Table 4). Collectively, these findings indicate that a reduction in sleep duration is associated with a graded increase in the risk of developing asthma.

**TABLE 4:** Relationship between nightly sleep duration and asthma in different models.

Characteristic	Statistics	Model 1	Model 2	Model 3
Nightly sleep duration				
Per SD increase		0.80 (0.72, 0.88) < 0.0001	0.83 (0.75, 0.92) 0.0002	0.83 (0.75, 0.92) 0.0003
Q0	1068 (15.78%)	Ref	Ref	Ref
Q1	2276 (33.62%)	0.66 (0.50, 0.87) 0.0029	0.72 (0.54, 0.96) 0.0230	0.74 (0.56, 0.98) 0.0362
Q2	1359 (20.07%)	0.48 (0.34, 0.67) < 0.0001	0.55 (0.39, 0.78) 0.0007	0.56 (0.40, 0.79) 0.0011
Q3	2067 (30.53%)	0.53 (0.40, 0.72) < 0.0001	0.58 (0.43, 0.79) 0.0004	0.59 (0.44, 0.79) 0.0005
P for trend		< 0.0001	0.0003	0.0004

### 3.4 Analysis of Threshold Effects and Model Robustness

**FIGURE 2:**
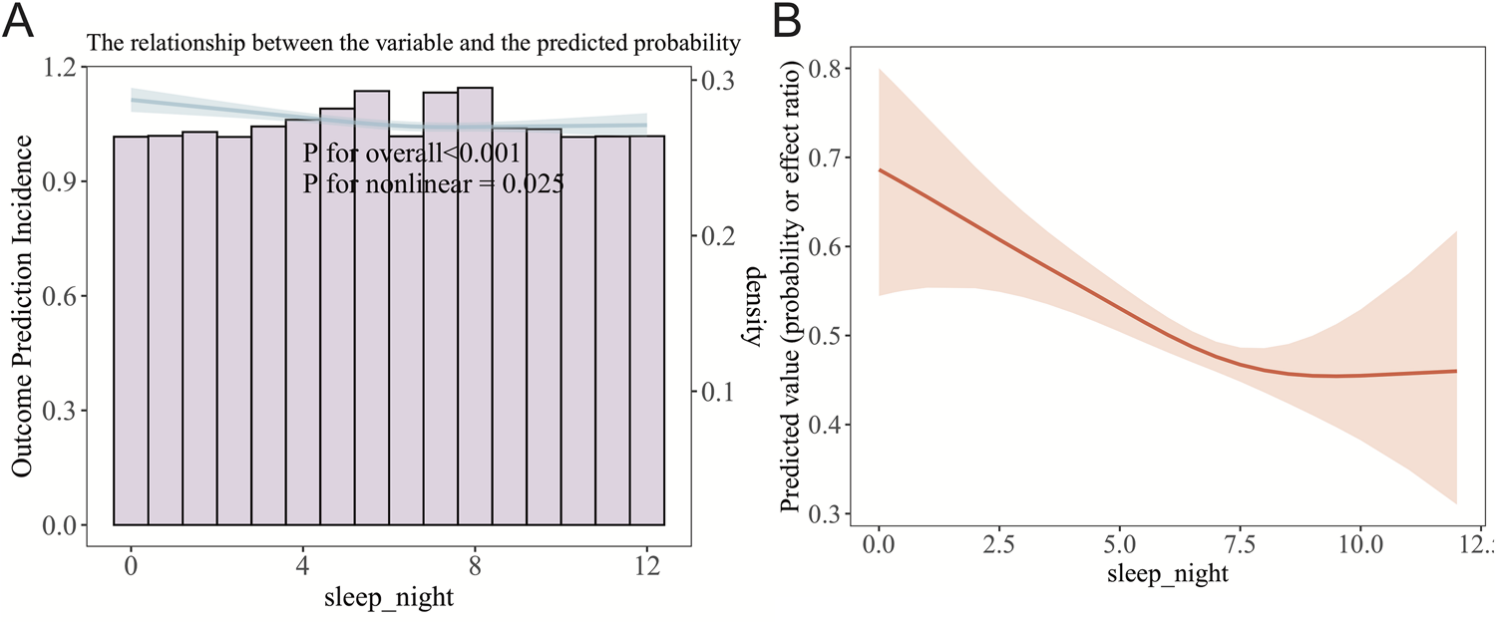
**(A)** The dose‐response curve as delineated by the RCS model and **(B)** The corresponding dose‐response curve as generated by GAM.

To further validate the association between sleep duration and asthma incidence, we conducted an RCS analysis and a corresponding threshold analysis. Our findings revealed a distinct J‐shaped relationship between sleep duration and the onset of asthma. We utilized a two‐piecewise Cox regression model to further investigate this relationship, and a threshold effect analysis identified an inflection point at 7.5 h of sleep (Figure 2A). Below this threshold, the risk of asthma escalated sharply with decreasing sleep duration, whereas above it, the risk plateaued. A subsequent likelihood ratio test yielded a significant result (*p* = 0.035), supporting the application of a piecewise model and confirming the non‐linear nature of the association. To bolster the robustness of these conclusions, sensitivity analyses were conducted using a GAM with smooth curve fitting. After adjusting for a comprehensive set of confounding variables—including age strata, educational attainment, urban/rural residence, marital status, use of clean cooking/heating fuel, and baseline C‐reactive protein levels—the significant non‐linear characteristic of the association between sleep duration and asthma risk persisted (Figure 2B), thereby substantiating the biological plausibility of the U‐shaped relationship.

### 3.5 Subgroup Analysis of the Association Between Sleep Duration and Asthma Risk

To explore potential heterogeneity in the association between sleep duration and asthma risk, subgroup analyses were conducted to examine effect modification by various population characteristics. The results indicated that the protective effect of sleep duration exhibited a significant dose‐response relationship across the majority of subgroups. A significant reduction in risk with prolonged sleep was observed in both males (P = 0.002) and females (P = 0.012); in smokers (P = 0.016) and non‐smokers (P = 0.002); and in both young and middle‐aged (P < 0.001) and elderly (P = 0.032) participants. This risk reduction was particularly pronounced among patients with diabetes (P = 0.009).Notably, the protective effect was more prominent among rural residents (P < 0.001) and individuals using clean cooking fuel (P < 0.001), whereas no significant trend was observed for urban residents (P = 0.311) or those in the non‐clean cooking fuel group (P = 0.091).Despite these differences in the magnitude of the trends across subgroups, formal tests for interaction did not reach statistical significance (all P for interaction > 0.05) (Table 5). This suggests that the protective effect of sleep duration is robust across diverse populations stratified by age, gender, metabolic state, and environmental exposures.

**TABLE 5:** Effect size of nightly sleep duration on asthma in pre‐specified and exploratory subgroups.

Characteristic	Q0	Q1	Q2	Q3	P for trend	P for interaction
Gender						0.926
Female	Ref	0.76 (0.52, 1.11)	0.59 (0.37, 0.94)	0.55 (0.37, 0.83)	0.002	
Male	Ref	0.68 (0.44, 1.05)	0.52 (0.31, 0.88)	0.58 (0.37, 0.91)	0.012	
Smoking status						0.582
No	Ref	0.75 (0.53, 1.06)	0.64 (0.42, 0.97)	0.58 (0.40, 0.83)	0.002	
Yes	Ref	0.71 (0.43, 1.17)	0.40 (0.21, 0.76)	0.58 (0.34, 0.97)	0.016	
Alcohol consumption						0.174
No	Ref	0.62 (0.44, 0.87)	0.57 (0.38, 0.85)	0.57 (0.40, 0.80)	0.001	
Yes	Ref	1.17 (0.68, 2.03)	0.62 (0.32, 1.22)	0.70 (0.39, 1.26)	0.074	
Hypertension						0.571
No	Ref	0.73 (0.52, 1.01)	0.53 (0.35, 0.80)	0.52 (0.36, 0.74)	< 0.001	
Yes	Ref	0.78 (0.45, 1.37)	0.64 (0.33, 1.24)	0.80 (0.46, 1.40)	0.388	
Diabetes						0.304
No	Ref	0.77 (0.57, 1.03)	0.60 (0.42, 0.85)	0.62 (0.46, 0.85)	< 0.001	
Yes	Ref	0.36 (0.10, 1.25)	0.17 (0.03, 0.83)	0.19 (0.04, 0.81)	0.009	
Residence						0.141
Urban	Ref	1.28 (0.71, 2.31)	0.71 (0.35, 1.47)	0.87 (0.46, 1.66)	0.311	
Rural	Ref	0.60 (0.43, 0.84)	0.53 (0.36, 0.79)	0.52 (0.37, 0.73)	< 0.001	
Clean fuel for heating						0.434
No	Ref	0.96 (0.50, 1.83)	0.93 (0.45, 1.90)	0.81 (0.41, 1.60)	0.505	
Yes	Ref	0.70 (0.51, 0.96)	0.47 (0.31, 0.71)	0.54 (0.39, 0.76)	< 0.001	
Clean fuel for cooking						0.941
No	Ref	0.82 (0.49, 1.38)	0.64 (0.35, 1.18)	0.65 (0.37, 1.14)	0.091	
Yes	Ref	0.69 (0.49, 0.98)	0.52 (0.34, 0.79)	0.56 (0.39, 0.80)	<0.001	
Age group						0.590
< 60 years	Ref	0.68 (0.44, 1.06)	0.48 (0.28, 0.80)	0.47 (0.29, 0.74)	< 0.001	
> 60 years	Ref	0.75 (0.51, 1.09)	0.62 (0.39, 0.99)	0.68 (0.46, 1.00)	0.032	

### 3.6 Genetic Correlation

Analyses of genetic correlation, employing both LDSC and HDL, elucidated a significant positive genetic correlation between nightly short sleep and asthma. The LDSC method yielded an estimated genetic correlation coefficient of 0.257 (SE = 0.034, *p* < 0.001), a finding corroborated by the HDL method, which returned a result of 0.247 (SE = 0.033, *p* < 0.001), thereby demonstrating a high degree of concordance between the two methodologies.

### 3.7 Identification and Enrichment Analysis of Pleiotropic Loci

**FIGURE 3:**
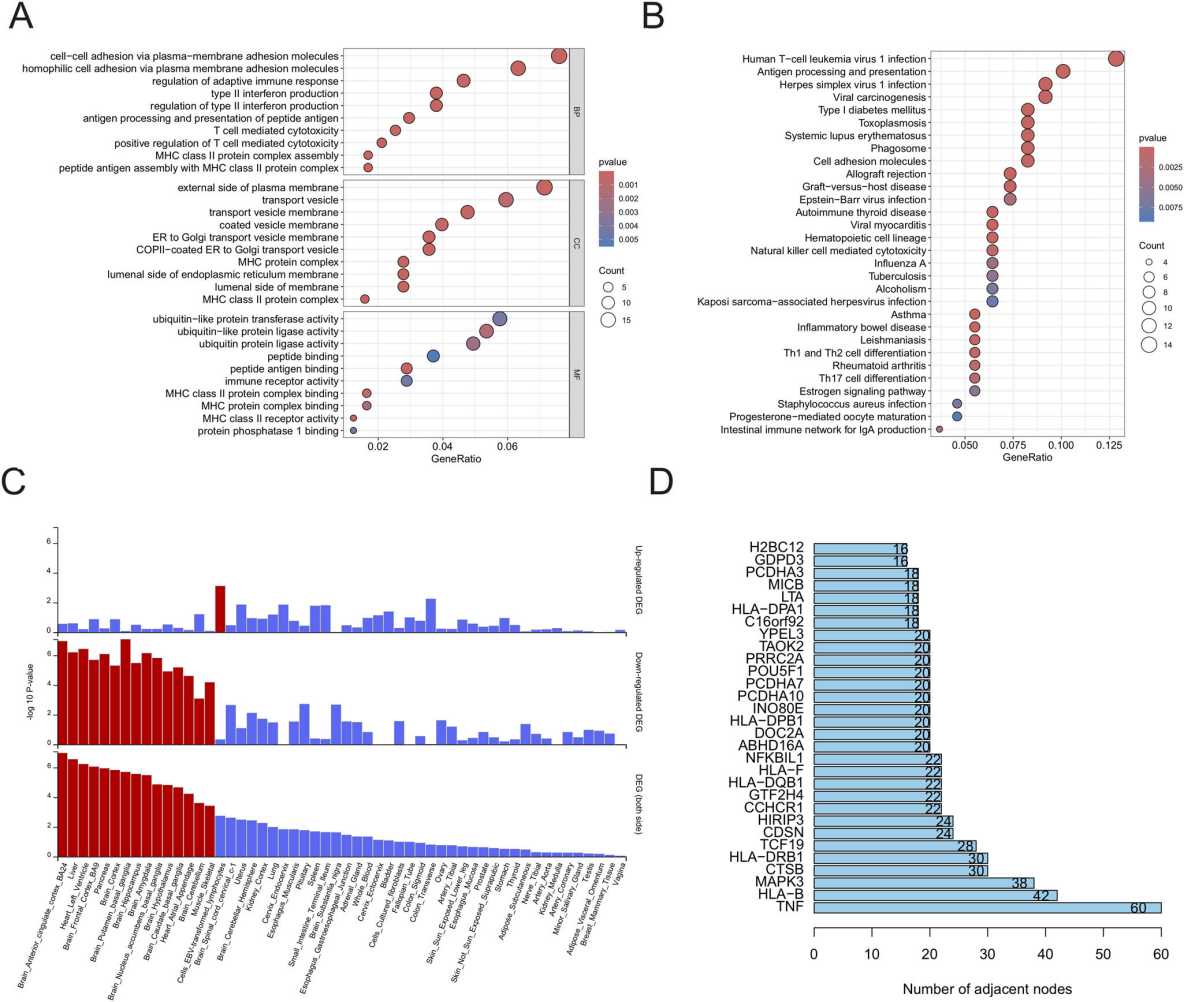
**(A)** GO enrichment analysis of pleiotropic genes, **(B)** KEGG pathway enrichment analysis of pleiotropic genes, **(C)** Tissue‐specific expression analysis of pleiotropic genes, and **(D)** Construction and analysis of the PPI network to identify central hub genes.

The Placo analysis identified 1364 SNPs as potential pleiotropic variants (Supplementary Table S1). Subsequent annotation of these loci using MAGMA delineated a set of 265 pleiotropic genes (Supplementary Table S2). To elucidate the underlying biological roles of this gene set, a GO enrichment analysis was performed, which revealed a significant functional convergence upon several key BP pertaining to the adaptive immune system.These processes prominently included antigen processing and presentation, such as antigen processing and presentation of peptide antigen and MHC class II protein complex assembly, alongside T‐cell mediated immune responses like T‐cell mediated cytotoxicity and its positive regulation, as well as the production of type II interferon. The enrichment extended to broader immunological functions, including the regulation of adaptive immune response and cell‐cell adhesion via plasma‐membrane adhesion molecules. Collectively, these findings underscore the pivotal role of this gene cohort in modulating T‐cell‐driven immunological pathways (Figure 3A), This functional profile was further corroborated by KEGG pathway analysis, which implicated these genes in pathways for autoimmune disorders, including autoimmune thyroid disease and systemic lupus erythematosus; infectious diseases, such as Epstein–Barr virus infection; and core immunological mechanisms like cell adhesion molecules and the phagosome (Figure 3B). The strong concordance between the GO and KEGG results jointly emphasizes the critical function of this pleiotropic gene set in mediating immune recognition, response, and the pathogenesis of immune‐related diseases.

Tissue‐specific enrichment analysis revealed that this gene set was significantly enriched within brain tissue, an observation notably absent in whole blood, lymphatic, and respiratory systems (Figure 3C). We therefore postulate that this cohort of brain‐enriched genes possesses a dual functionality. On one hand, they may serve as integral components of the molecular networks governing the sleep‐wake cycle, thereby directly contributing to the maintenance of sleep homeostasis. On the other hand, and perhaps more critically, they may represent crucial nodes for the central nervous system's regulation of peripheral immunity.

### 3.8 Construction and Analysis of the Protein‐Protein Interaction Network

To elucidate the pivotal regulatory elements within the system, a PPI network was constructed and subsequently subjected to a topological analysis designed to identify central “hub” genes. Within this network, the gene YPEL3 was distinguished by a notable degree of connectivity (degree = 20) (Figure 3D). This finding suggests that YPEL3 functions not as an isolated entity but as a highly interconnected node, implying its profound integration and potential functional importance within the broader interaction landscape.

### 3.9 Identification of Causal Pleiotropic Loci

The 1367 pleiotropic loci initially identified by the PLACO analysis were subjected to a linkage disequilibrium clumping procedure using the PLINK software, which resolved these signals into 38 independent SNPs. Among these independent loci, three garnered particular attention due to their potential shared causality between nightly short sleep and asthma pathogenesis: rs205024 (PPH4 = 0.522), rs6939576 (PPH4 = 0.996), and rs13107325 (PPH4 = 0.927) (Supplementary Table S3). Subsequent functional annotation revealed distinct mechanistic profiles for these variants. The SNP rs13107325 presented a CADD score of 24.2, ranking it within the top 1% of putatively deleterious variants in the human genome and implying a high likelihood of functional detriment. As a missense variant, its predicted pathogenicity stems from an alteration to the amino acid sequence of the protein encoded by the SLC39A8 gene, thereby potentially compromising its normative function. In concordance with a non‐regulatory mechanism, its RegulomeDB score of 6 indicates minimal evidence for involvement in transcriptional regulation.

Conversely, while rs205024 exhibited a more modest posterior probability of a shared effect, its notable CADD score (10.25) combined with a high‐confidence RegulomeDB score (1f) suggests a different functional modality. Rather than altering a protein sequence, this locus is predicted to exert its influence by modulating the binding affinity of a specific transcription factor, which in turn regulates the expression of a downstream target gene.

### 3.10 Identification of Causal Pleiotropic Genes

**FIGURE 4:**
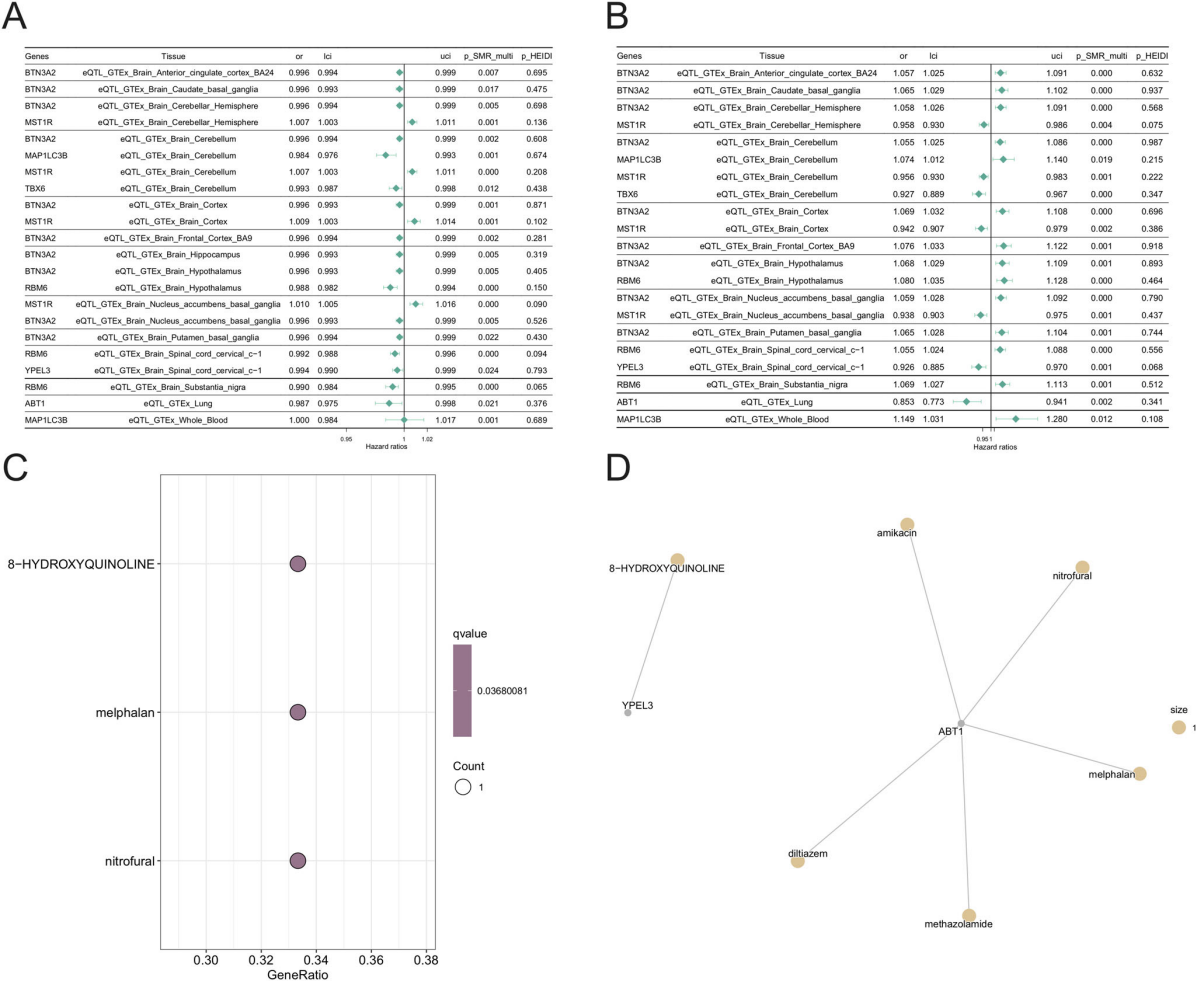
**(A)** The SMR results for the association between the expression levels of pleiotropic genes and nightly short sleep, **(B)** The SMR results for the association between the expression levels of pleiotropic genes and asthma, **(C)** Drug enrichment analysis of GO enrichment analysis of cause pleiotropic genes, and **(D)** A network plot showing the interactions between drug enrichment analysis of cause pleiotropic genes and the identified potential drugs.

Subsequent to the SMR analysis of nightly short sleep and asthma, our findings revealed a cohort of significant genes. Six of these—BTN3A2, MST1R, RBM6, ABT1, TBX6, and YPEL3—exhibited a significant causal influence (Psmr < 0.05, PHEIDI > 0.05) on both phenotypes, although this effect was confined to a single tissue type. Furthermore, two genes, MAP1LC3B and TBX6, demonstrated this significant causal link across two distinct tissue types. Our subsequent objective was to delineate which of these genes possessed a concordant direction of effect on both nightly short sleep and asthma within the same tissue context. This directional analysis revealed that three genes, namely TBX6, ABT1, and YPEL3, exerted a consistently negative effect. In contrast, MAP1LC3B displayed antagonistic effects across different tissues. Accordingly, based on their consistent and directionally harmonious causal influence, TBX6, ABT1, and YPEL3 were designated as high‐priority, independent drug targets for investigating the comorbidity of nightly short sleep and asthma (Figures 4A,B).

### 3.11 Prediction of Candidate Therapeutics

An enrichment analysis of the DSigDB database identified 8‐hydroxyquinoline, melphalan, and nitrofurazone as the three candidate compounds most significantly associated with the shared pathophysiology of nightly short sleep and asthma (Figures 4C,D). The therapeutic targets of these agents were found to involve the genes YPEL3 and ABT1. Upon consideration of crucial factors such as blood‐brain barrier permeability and established safety profiles, 8‐hydroxyquinoline emerged as the most compelling candidate. Characterized as a “privileged structure” with known central nervous system penetrance, this molecule is known to possess a spectrum of biological activities, including metal chelation, neuroprotection, and anti‐neoplastic properties.

### 3.12 Mendelian Randomization

Applying stringent criteria (*r* ^2^ < 0.001, *p* < 5 × 10^−8^), a total of 24 SNPs associated with nightly short sleep were initially identified. To preclude the influence of confounding variables, we conducted a comprehensive search of the NHGRI‐EBI Catalog database to screen for and exclude any SNPs significantly linked to confounding factors such as BMI, smoking, alcohol consumption, depression, or asthma (*p* < 5 × 10^−8^). This filtering process revealed that rs2517827 exhibited a significant association with BMI; rs13107325 demonstrated pronounced associations with BMI, smoking, alcohol consumption, and asthma; and rs11763750 was significantly correlated with depressive states. Subsequently, the remaining 21 SNPs were cross‐referenced with the 1367 pleiotropic loci identified by PLACO, which led to the identification of an additional pleiotropic site, rs205024. Following the exclusion of these four SNPs due to their confounding or pleiotropic effects, we employed five distinct MR methodologies to evaluate the causal relationship between nightly short sleep and asthma. The IVW method constituted the principal analytical approach, supplemented by MR‐Egger, weighted median, and other sensitivity analyses.

**FIGURE 5:**
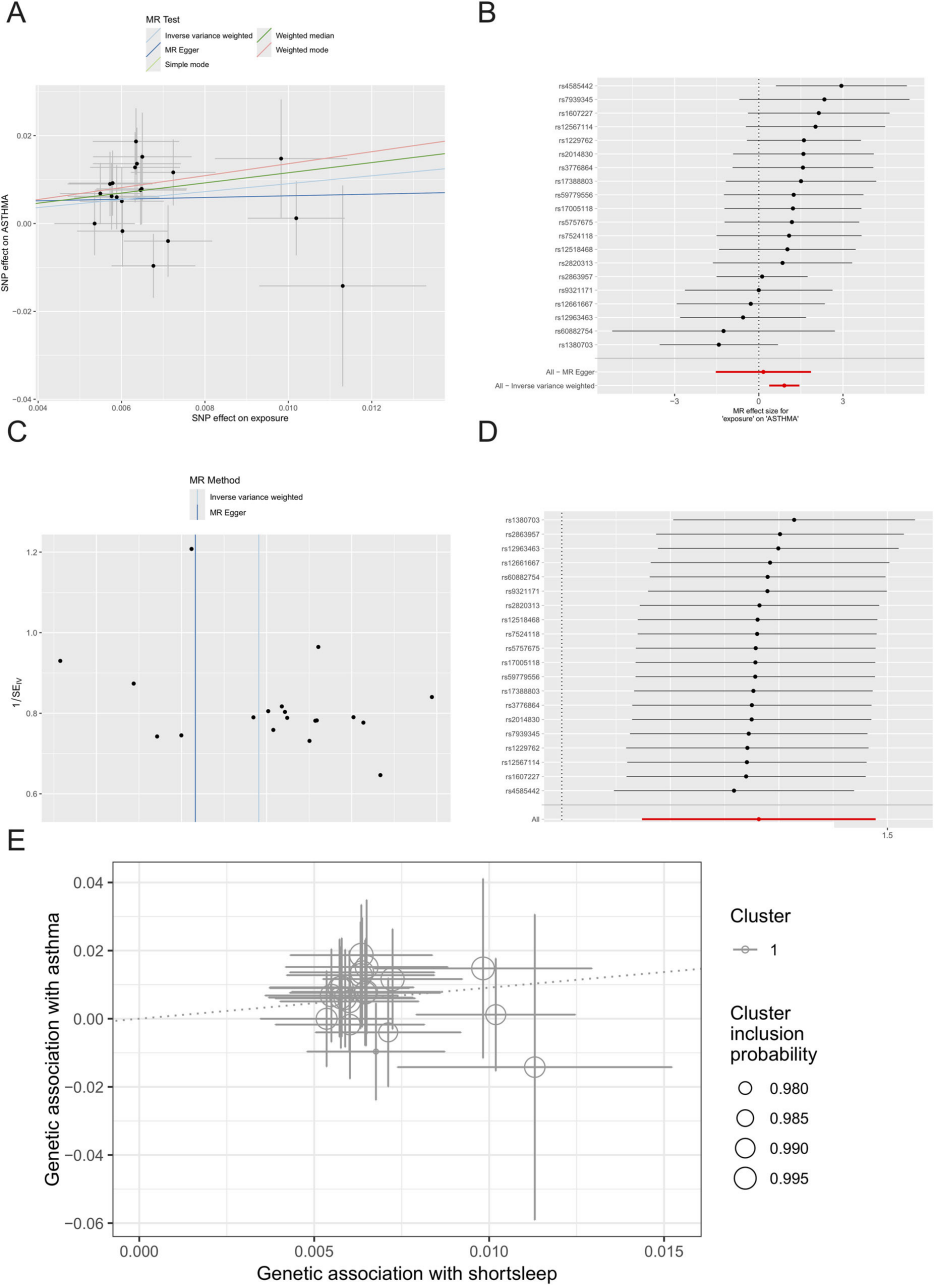
**(A)** A scatter plot depicting the effect sizes of individual instrumental SNPs on the exposure versus their corresponding effects on the outcome. The slope of the regression line represents the overall causal estimate, **(B)** A forest plot providing a comparative visualization of the causal estimates derived from five distinct MR methodologies, **(C)** A funnel plot designed to assess heterogeneity and directional pleiotropy, **(D)** A leave‐one‐out sensitivity analysis plot, which systematically recalculates the causal estimate by omitting one SNP at a time, thereby evaluating the influence of individual variants on the overall result, and **(E)** A scatter plot generated by the MR‐Clust algorithm to identify potential clusters among the genetic instruments.

The *F*‐statistics for all instrumental variables (IVs) in this analysis surpassed the conventional threshold of 10, indicating an absence of weak instrument bias and thereby confirming the robustness of our findings (Supplementary Table S4). The primary IVW analysis demonstrated a significant positive association between genetically predicted nightly short sleep and the risk of asthma (OR, 2.478; 95% CI: [1.447–4.245]; *p* = 0.00095), the influence of individual SNP loci on asthma is graphically illustrated in Figures 5A and 5B. Furthermore, Cochran's *Q* test indicated no significant heterogeneity among the instruments (P = 0.622). The MR‐Egger intercept analysis did not suggest the presence of directional horizontal pleiotropy (P = 0.272), a finding corroborated by the MR‐PRESSO test, which also failed to detect pleiotropic outliers (P = 0.640) (Supplementary Table S5), while the funnel plot demonstrated symmetrical distribution of effect estimates, reinforcing the absence of systematic bias (Figure 5C). Leave‐one‐out sensitivity analyses were employed to scrutinize the influence of individual SNPs on the causal inference. Iterative removal of each SNP and subsequent MR replications yielded no substantial alteration in the observed association (Figure 5D). These results collectively imply that the instrumental variables do not exert a significant effect on the outcome through pathways independent of the exposure. Finally, a Steiger test was performed to ascertain the directionality of this relationship, and its results did not support a reverse causal effect from asthma on nightly short sleep (*p* < 0.001) (Supplementary Table S6).

### 3.13 MR‐Clust Analysis of Nightly Short Sleep on Asthma

To conduct a more profound investigation into potential pleiotropy, a cluster analysis was subsequently performed utilizing the MR‐Clust methodology. The results revealed that all 20 instrumental variables associated with short nocturnal sleep were consolidated within a single, unified cluster. Critically, no other independent functional clusters were observed, which indicates that there is insufficient evidence to support the existence of divergent causal pathways arising from pleiotropic effects.

### 3.14 MRcML

Analysis using the MRcML method revealed a significant, positive causal effect of nightly short sleep on asthma. The optimal model, selected based on the Bayesian Information Criterion (BIC), yielded an effect estimate of *β* = 0.927 (SE = 0.280, *p* = 0.0009) and did not identify any potentially invalid instrumental variables. The model‐averaged result (MA‐BIC) corroborated this finding (*β* = 0.930, SE = 0.281, *p* = 0.0009). Although an alternative model based on the Akaike Information Criterion (AIC) detected three invalid instruments and produced a more conservative estimate (*β* = 0.969, SE = 0.297, *p* = 0.001), it nevertheless supported the conclusion that nightly short sleep has a deleterious effect on asthma. The robustness of the results under the BIC framework and the general consistency across models indicate a stable causal effect of the exposure on the outcome (Supplementary Table S7).

### 3.15 ConMix

The ConMix analysis clearly shows that nightly short sleep causally increases asthma risk (*β* = 1.585, OR = 4.881, *p* = 0.0006), meaning individuals with nightly short sleep face nearly five times higher odds of developing asthma compared to those with adequate sleep. By employing its unique contamination mixture model, the ConMix method effectively accounts for potential confounding from horizontal pleiotropy, lending greater credence to this causal inference compared to traditional Mendelian randomization analyses.

### 3.16 CAUSE

**FIGURE 6:**
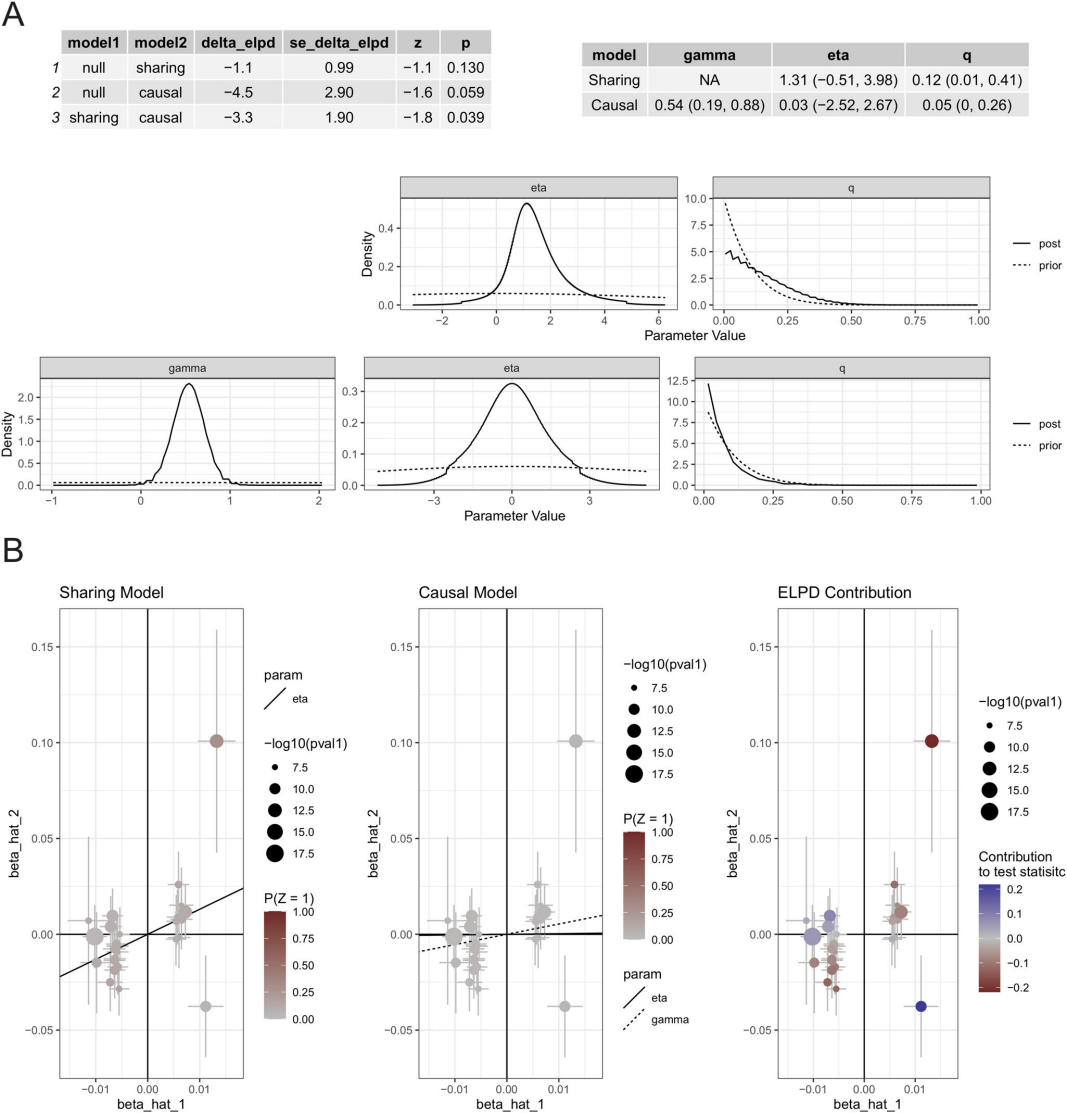
**(A)** Parameter posterior distributions and **(B)** Effect size scatter plot and model fit.

An analysis utilizing the CAUSE methodology yielded a negative expected log pointwise posterior density difference (ΔELPD) of −3.3. This outcome indicates a superior statistical fit for the causal model in comparison to the sharing model, which presumes only pleiotropy without a direct causal relationship. Furthermore, the CAUSE analysis provided additional evidence substantiating that nightly short sleep increases the risk of developing asthma, estimating a positive causal effect (*γ* = 0.54) (Figures 6A–B).

## 4 Discussion

While the comorbidity of nightly short sleep and asthma is well‐recognized, the existence of a direct causal relationship and its underlying biological mechanisms have remained largely unelucidated. The present investigation, leveraging large‐scale genomic data and integrating a suite of advanced genetic analytical methodologies, not only provides the first systematic evidence for a significant causal effect of nightly short sleep on asthma but also uncovers an extensive and profound genetic commonality between the two conditions. Our subsequent analyses indicate that these shared risk genes do not operate in isolation; rather, they constitute an intricate regulatory network that spans multiple tiers of biological organization. At the systems level, this network concurrently influences both the maintenance of sleep homeostasis and the pathophysiology of asthma through neuro‐regulatory pathways. At the molecular level, we discovered that these same genes synergistically orchestrate canonical inflammatory and immune activation pathways.

This synergistic regulation thereby establishes a molecular bridge linking central nervous system dysregulation of sleep with peripheral airway inflammation, thus offering a novel perspective for a fundamental understanding of their mechanism of comorbidity. It has been established that nightly short sleep induces a state of systemic, low‐grade inflammation, a mechanism plausibly linked to the onset and exacerbation of asthma. A prospective cohort study in asthma patients found that shorter sleep duration was significantly correlated with aggravated airway inflammation, manifested as elevated levels of interleukin‐6 (IL‐6) and tumor necrosis factor‐alpha (TNF‐*α*) in sputum (Irwin et al. 2006, Mullington et al. 2010). Furthermore, even a single night of mild sleep deprivation has been shown to significantly increase serum levels of IL‐6 and TNF‐*α*, along with their corresponding mRNA expression, indicating that sleep loss can rapidly activate pro‐inflammatory pathways. The immunopathology of asthma involves the overproduction of various cytokines, classically characterized by Type 2 inflammation driven by IL‐4, IL‐5, and IL‐13 released from eosinophils, mast cells, and Th2 cells, or in severe asthma, by Type 1 inflammation mediated by IFN‐*γ* and CXCL10 (Lambrecht et al. 2015). Nightly short sleep impairs the immune system through multiple mechanisms, including reducing lymphocyte counts, suppressing T‐cell proliferation, and downregulating the expression of human leukocyte antigen‐DR (Besedovsky et al. 2012). In allergic mouse models, sleep deprivation has been shown to induce neutrophilic inflammation via the Th17 pathway and to result in corticosteroid resistance (Hu et al. 2022). Moreover, nightly short sleep can disrupt the equilibrium of T‐helper cell subsets and alter the levels of cytokines such as IL‐2, IL‐4, and IFN‐*γ* (Hu et al. 2022, Besedovsky et al. 2019). This immunological dysregulation may render individuals with asthma more susceptible to allergens, a notion supported by findings of a dose‐response relationship between nightly short sleep, an increased risk of food allergies, and a greater number of positive skin prick tests. Notably, nightly short sleep also heightens susceptibility to infections, which are primary triggers for acute asthma exacerbations. This establishes a pernicious cycle: insufficient sleep compromises immunological defenses, leading to more severe respiratory infections that, in turn, provoke a worsening of asthma symptoms. Additionally, studies in mouse models have demonstrated that nightly short sleep induces neutrophilic lung inflammation via the IL‐17 signaling pathway, a process accompanied by corticosteroid resistance, suggesting that sleep deprivation may adversely affect therapeutic responsiveness in asthma (Nunes et al. 2018).

A particularly compelling and thought‐provoking finding from this study emerged from the tissue‐specific enrichment analysis. This analysis revealed that a cohort of genes with well‐defined immunological functions exhibited significant expression enrichment within brain tissue—an enrichment conspicuously absent from traditional immune‐related tissues such as whole blood, the lymphatic system, and the respiratory system. To reconcile this apparent paradox, we posit a central hypothesis: that this brain‐enriched gene set possesses a “dual functionality,” constituting a molecular bridge between the central nervous system and the peripheral immune system. We propose the following mechanistic framework for this hypothesis. On one hand, as integral parts of the brain's molecular architecture, these genes may be directly involved in the homeostatic regulation of the sleep‐wake cycle. On the other hand, and perhaps more critically, they may serve as pivotal nodes in the central regulation of peripheral immunity. In a state of physiological homeostasis, the central nervous system likely maintains peripheral immune equilibrium through pathways associated with these genes. However, upon exposure to stressors such as sleep deprivation or circadian disruption, the resultant disequilibrium within the CNS is transduced to the periphery via altered signaling from neuroendocrine and autonomic nervous pathways. This perturbation of central signaling, in turn, precipitates peripheral immune dysregulation, which within the respiratory system may manifest as a reduced threshold for, or an augmented intensity of, airway inflammation. Such a mechanism would heighten host susceptibility to immune‐mediated disorders like asthma and potentially exacerbate their pathophysiology.

This study first suggested a comorbidity between nocturnal nightly short sleep and asthma through epidemiological investigation and subsequently elucidated their genetic correlation and overlap through genetic analysis. We demonstrated that their shared pathogenic genes converge upon canonical inflammatory response and immune activation signaling pathways. Furthermore, through Mendelian randomization analysis, we validated nightly short sleep as a potential risk factor for asthma, thereby surmounting the limitations inherent in observational studies and mitigating bias from extraneous variables. This work furnishes a novel scientific basis and holds clinical value for understanding the symptomatology of asthma and for preventing its onset. Although our use of GWAS summary data derived from European populations may constrain the generalizability of our findings, parallel observations of comorbidity in Chinese populations suggest that our results may possess broader applicability. Nevertheless, the present study is not without its limitations. Future investigations should therefore consider employing large‐scale cohort studies to quantify the reciprocal risk of transition between nightly short sleep and asthma and to further delineate their shared molecular mechanisms.

## 5 Conclusion

In conclusion, the present investigation has not only delineated a cohort of pleiotropic genes that form a nexus between sleep and immune disorders but, more critically, has offered a novel molecular perspective on the clinical observation that sleep disturbances exacerbate immunological conditions by revealing the specific enrichment of these genes within brain tissue. Our “dual function” hypothesis postulates that the brain leverages this particular set of “immune genes” as intermediaries to translate circadian and sleep dysregulation into peripheral immune dysfunction. This framework, therefore, unveils promising new targets for the future comprehension of, and therapeutic intervention in, these interconnected pathologies.

## Author Contributions

Y.‐P.Z. designed the research study. C.F. performed the research and analyzed the data. Y.‐P.Z., W.‐X., and C.F. wrote the manuscript. All authors contributed to editorial changes in the manuscript. All authors read and approved the final manuscript.

## Ethics Statement

Most of the data in this study were publicly available and nonidentifiable. Therefore, no ethical approval was required to access it.

## Conflicts of Interest

The authors declare that the research was conducted in the absence of any commercial or financial relationships that could be construed as a potential conflict of interest.

## Peer Review

The peer review history for this article is available at https://publons.com/publon/10.1002/brb3.71001

## Supporting information




**Supporting Table:1** Significant loci for the PLACO phenotype identified through the Genome‐Wide Association Study
**Supporting Table:2** Significant genes associated with PLACO identified via MAGMA gene‐based analysis
**Supporting Table:3** Colocalization results for nightly short sleep and asthma
**Supporting Table:4** Characteristics of the instrumental variables (SNPs) used in the Mendelian randomization analysis
**Supporting Table:5** Results of the Mendelian Randomization Analysis for Nightly Short Sleep and Asthma
**Supporting Table:6** Causal directionality test for the Mendelian randomization analysis
**Supporting Table:7** Results from cML‐MR analysis

## Data Availability

The data generated or analyzed during the current study available from corresponding author on reasonable request.

## References

[brb371001-bib-0001] Besedovsky, L. , T. Lange , and J. Born . 2012. “Sleep and Immune Function.” Pflugers Archiv: European Journal of Physiology 463, no. 1: 121–137. 22071480 10.1007/s00424-011-1044-0 PMC3256323

[brb371001-bib-0002] Besedovsky, L. , T. Lange , and M. Haack . 2019. “Sleep‐Immune Crosstalk in Health and Disease.” Physiological Reviews 99, no. 3: 1325–1380. 30920354 10.1152/physrev.00010.2018 PMC6689741

[brb371001-bib-0003] Bulik‐Sullivan, B. , H. K. Finucane , V. Anttila , et al. 2015. “An Atlas of Genetic Correlations Across Human Diseases and Traits.” Nature Genetics 47, no. 11: 1236–1241. 26414676 10.1038/ng.3406 PMC4797329

[brb371001-bib-0004] Burgess, S. , C. N. Foley , E. Allara , J. R. Staley , and J. M. M. Howson . 2020. “A Robust and Efficient Method for Mendelian Randomization With Hundreds of Genetic Variants.” Nature Communications 11, no. 1: 376. 10.1038/s41467-019-14156-4 PMC6969055 31953392

[brb371001-bib-0005] Burgess, S. , and S. G. Thompson CRP CHD Genetics Collaboration . 2011. “Avoiding Bias From Weak Instruments in Mendelian Randomization Studies.” International Journal of Epidemiology 40, no. 3: 755–764. 21414999 10.1093/ije/dyr036

[brb371001-bib-0006] Davies, N. M. , M. V. Holmes , and G. Davey Smith . 2018. “Reading Mendelian Randomisation Studies: A Guide, Glossary, and Checklist for Clinicians.” BMJ (Clinical Research Edition) 362: k601. 10.1136/bmj.k601 PMC6041728 30002074

[brb371001-bib-0007] Foley, C. N. , A. M. Mason , P. D. Kirk , and S. Burgess . 2021. “MR‐Clust: Clustering of Genetic Variants in Mendelian Randomization With Similar Causal Estimates.” Bioinformatics 37, no. 4: 531–541. 32915962 10.1093/bioinformatics/btaa778 PMC8088327

[brb371001-bib-0008] Giambartolomei, C. , D. Vukcevic , E. E. Schadt , et al. 2014. “Bayesian Test for Colocalisation Between Pairs of Genetic Association Studies Using Summary Statistics.” PLoS Genetics 10, no. 5: e1004383. 24830394 10.1371/journal.pgen.1004383 PMC4022491

[brb371001-bib-0009] Groenewoud, D. , A. Shye , and R. Elkon . 2022. “Incorporating Regulatory Interactions Into Gene‐set Analyses for GWAS Data: A Controlled Analysis With the MAGMA Tool.” PLoS Computational Biology 18, no. 3: e1009908. 35316269 10.1371/journal.pcbi.1009908 PMC8939811

[brb371001-bib-0010] Hemani, G. , J. Zheng , B. Elsworth , et al. 2018. “The MR‐Base Platform Supports Systematic Causal Inference Across the Human Phenome.” ELife 7: e34408. 29846171 10.7554/eLife.34408 PMC5976434

[brb371001-bib-0011] Hu, Z. , X. Song , and K. Hu . 2022. “The Effect of Short Sleep Duration on the Development of Asthma.” International Journal of Clinical Practice 2022, no. 1: 3378821. 35685599 10.1155/2022/3378821 PMC9159162

[brb371001-bib-0012] Huang, K. , T. Yang , J. Xu , et al. 2019. “Prevalence, Risk Factors, and Management of Asthma in China: A National Cross‐Sectional Study.” The Lancet 394, no. 10196: 407–418. 10.1016/S0140-6736(19)31147-X 31230828

[brb371001-bib-0013] Irwin, M. R. , M. Wang , C. O. Campomayor , A. Collado‐Hidalgo , and S. Cole . 2006. “Sleep Deprivation and Activation of Morning Levels of Cellular and Genomic Markers of Inflammation.” Archives of Internal Medicine 166, no. 16: 1756–1762. 16983055 10.1001/archinte.166.16.1756

[brb371001-bib-0014] Kircher, M. , D. M. Witten , P. Jain , O.'r. BJ , and G. M. Cooper . 2014. “A General Framework for Estimating the Relative Pathogenicity of Human Genetic Variants.” Nature Genetics 46, no. 3: 310–315. 24487276 10.1038/ng.2892 PMC3992975

[brb371001-bib-0015] Lambrecht, B. N. , and H. J. N. Hammadi . 2015. “The Immunology of Asthma.” Nature Immunology 16, no. 1: 45–56. 25521684 10.1038/ni.3049

[brb371001-bib-0016] Morrison, J. , N. Knoblauch , J. H. Marcus , M. Stephens , and X. He . 2020. “Mendelian Randomization Accounting for Correlated and Uncorrelated Pleiotropic Effects Using Genome‐wide Summary Statistics.” Nature Genetics 52, no. 7: 740–747. 32451458 10.1038/s41588-020-0631-4 PMC7343608

[brb371001-bib-0017] Mullington, J. M. , N. S. Simpson , H. K. Meier‐Ewert , and M. Haack . 2010. “Sleep Loss and Inflammation.” Best Practice & Research Clinical Endocrinology & Metabolism 24, no. 5: 775–784. 21112025 10.1016/j.beem.2010.08.014 PMC3548567

[brb371001-bib-0018] Ni, X. , X. Li , and J. Li . 2025. “Insomnia Associated With Increased Risk of Atopic Dermatitis: A Two‐Sample Mendelian Randomization Study.” Brain and Behavior 15, no. 5: e70512. 40320904 10.1002/brb3.70512 PMC12050649

[brb371001-bib-0019] Ning, Z. , Y. Pawitan , and S. Shen . 2020. “High‐Definition Likelihood Inference of Genetic Correlations Across Human Complex Traits.” Nature Genetics 52, no. 8: 859–864. 32601477 10.1038/s41588-020-0653-y

[brb371001-bib-0020] Nunes, J. O. , J. de Souza Apostolico , D. A. Andrade , et al. 2018. “Sleep Deprivation Predisposes Allergic Mice to Neutrophilic Lung Inflammation.” The Journal of Allergy and Clinical Immunology 141, no. 3: 1018–1027. 28732645 10.1016/j.jaci.2017.06.025

[brb371001-bib-0021] Purcell, S. , B. Neale , K. Todd‐Brown , et al. 2007. “PLINK: A Tool Set for Whole‐genome Association and Population‐based Linkage Analyses.” American Journal of Human Genetics 81, no. 3: 559–575. 17701901 10.1086/519795 PMC1950838

[brb371001-bib-0022] Ray, D. , and N. Chatterjee . 2020. “A Powerful Method for Pleiotropic Analysis Under Composite Null Hypothesis Identifies Novel Shared Loci Between Type 2 Diabetes and Prostate Cancer.” PLoS Genetics 16, no. 12: e1009218. 33290408 10.1371/journal.pgen.1009218 PMC7748289

[brb371001-bib-0023] Shin, Y. H. , J. Hwang , R. Kwon , et al. 2023. “Global, Regional, and National Burden of Allergic Disorders and Their Risk Factors in 204 Countries and Territories, From 1990 to 2019: A Systematic Analysis for the Global Burden of Disease Study 2019.” Allergy 78, no. 8: 2232–2254. 37431853 10.1111/all.15807 PMC10529296

[brb371001-bib-0024] Song, K. J. 2023. “Integration of the Drug‐Gene Interaction Database (DGIdb 4.0) With Open Crowdsource Efforts.” Nucleic Acids Research 49, no. D1: D1144–D1151. 10.1093/nar/gkaa1084 PMC7778926 33237278

[brb371001-bib-0025] Verbanck, M. , C.‐Y. Chen , B. Neale , and R. Do . 2018. “Detection of Widespread Horizontal Pleiotropy in Causal Relationships Inferred From Mendelian Randomization Between Complex Traits and Diseases.” Nature Genetics 50, no. 5: 693–698. 29686387 10.1038/s41588-018-0099-7 PMC6083837

[brb371001-bib-0026] Xue, H. , X. Shen , and W. Pan . 2021. “Constrained Maximum Likelihood‐based Mendelian Randomization Robust to Both Correlated and Uncorrelated Pleiotropic Effects.” American Journal of Human Genetics 108, no. 7: 1251–1269. 34214446 10.1016/j.ajhg.2021.05.014 PMC8322939

[brb371001-bib-0027] Yang, G. , Y.‐Y. Han , T. Sun , et al. 2019. “Sleep Duration, Current Asthma, and Lung Function in a Nationwide Study of US Adults.” American Journal of Respiratory and Critical Care Medicine 200, no. 7: 926–929. 31225970 10.1164/rccm.201905-1004LE PMC6812440

[brb371001-bib-0028] Zhu, Z. , F. Zhang , H. Hu , et al. 2016. “Integration of Summary Data From GWAS and eqtl Studies Predicts Complex Trait Gene Targets.” Nature Genetics 48, no. 5: 481–487. 27019110 10.1038/ng.3538

